# Green Conversion of Agroindustrial Wastes into Chitin and Chitosan by *Rhizopus arrhizus* and *Cunninghamella elegans* Strains

**DOI:** 10.3390/ijms15059082

**Published:** 2014-05-21

**Authors:** Lúcia Raquel Ramos Berger, Thayza Christina Montenegro Stamford, Thatiana Montenegro Stamford-Arnaud, Sergio Roberto Cabral de Alcântara, Antonio Cardoso da Silva, Adamares Marques da Silva, Aline Elesbão do Nascimento, Galba Maria de Campos-Takaki

**Affiliations:** 1 Post-Graduation Program in Biological Sciences, Federal University of Pernambuco, Recife, PE 50670-420, Brazil; E-Mails: quelberger@hotmail.com (L.R.R.B.); adamaresmarques@hotmail.com (A.M.S.); 2 Nucleus of Research in Environmental Science and Biotechnology (NPCIAMB), Catholic University of Pernambuco, Recife, PE 50050-590, Brazil; E-Mails: antoniocardoso2000@yahoo.com.br (A.C.S.); elesbao@unicap.br (A.E.N.); 3 Department of Tropical Medicine, Center of Health Sciences, Federal University of Pernambuco, Recife, PE 50670-420, Brazil; E-Mails: thayzastamford@yahoo.com.br (T.C.M.S.); thatianaarnaud@hotmail.com (T.M.S.-A.); 4 Post-Graduation Program in Development and Environment, Federal University of Paraíba, Campus 1, João Pessoa, PB 58051-900, Brazil; E-Mail: pohlux@gmail.com

**Keywords:** Zygomycetes, polymer, agroindustrial waste, antibacterial activity

## Abstract

This article sets out a method for producing chitin and chitosan by *Cunninghamella elegans* and *Rhizopus arrhizus* strains using a green metabolic conversion of agroindustrial wastes (corn steep liquor and molasses). The physicochemical characteristics of the biopolymers and antimicrobial activity are described. Chitin and chitosan were extracted by alkali-acid treatment, and characterized by infrared spectroscopy, viscosity and X-ray diffraction. The effectiveness of chitosan from *C. elegans* and *R. arrhizus* in inhibiting the growth of *Listeria monocytogenes*, *Staphylococcus aureus*, *Pseudomonas aeruginosa*, *Salmonella enterica*, *Escherichia coli* and *Yersinia enterocolitica* were evaluated by determining the minimum inhibitory concentrations (MIC) and the minimum bactericidal concentrations (MBC). The highest production of biomass (24.60 g/L), chitin (83.20 mg/g) and chitosan (49.31 mg/g) was obtained by *R. arrhizus*. Chitin and chitosan from both fungi showed a similar degree of deacetylation, respectively of 25% and 82%, crystallinity indices of 33.80% and 32.80% for chitin, and 20.30% and 17.80% for chitosan. Both chitin and chitosan presented similar viscosimetry of 3.79–3.40 cP and low molecular weight of 5.08 × 10^3^ and 4.68 × 10^3^ g/mol. They both showed identical MIC and MBC for all bacteria assayed. These results suggest that: agricultural wastes can be produced in an environmentally friendly way; chitin and chitosan can be produced economically; and that chitosan has antimicrobial potential against pathogenic bacteria.

## 1. Introduction

Chitosan is a natural co-polymer of chitin, comprising units of 2-amino-2-desoxi-d-glycopyranose and of 2-acetamide-2-desoxi-d-glycopyranose interconnected by glycosidic bonds β-1.4 in variable proportions. The first type of unit is frequently present in chitosan [1]. Chitin is present as a structural element in the exoskeleton of crustaceans, mollusks, annelids, coelenterates and insects. It is also a major component of the fungal cell wall, particularly of Zygomycetes [2,3]. Chitosan is a cationic and linear polymer, naturally found in the cell wall of fungi, mainly in the Mucorales order. Although the main commercial source of chitosan is crustacean shells, some studies have proposed that cultivating selected fungi could provide an effective source of chitosan for industrial applications [1,2,4].

Recent advances in fermentation technology for the fungal production of chitin and chitosan have received worldwide attention and some studies suggest that many of the problems encountered when extracting biopolymers in a traditional way can be overcome [5,6]. The use of chitin and chitosan from fungi biomass has great advantages, such as independence from seasonal factors, wide scale production, simultaneous extraction of the polymers, and the fact that the process of extracting chitosan from the biomass of fungi is simple and cheap, resulting in reductions in the time and cost of production. Moreover, this strategy avoids protein contamination, particularly from proteins that could cause allergic reactions in individuals with shellfish allergies [4,7,8].

The biowaste from some industrial by-products such as molasses, corn steep liquor and cassava wastewater can be used as very economical nutritional sources when cultivating fungi. This alternative favors obtaining a byproduct with high added value as well as decreasing total production costs [9,10]. Molasses from cane sugar, a byproduct of the sugar industry, has a large amount of fermentable sugar and is considered a waste that is easy to handle, costs little and has great potential and many applications at an industrial level. By virtue of its composition, molasses is used mainly as a source of carbon and energy but it is necessary to supplement it with nitrogen and some minerals, especially phosphorus and magnesium [11]. Corn steep liquor, a residue from the corn processing industry, has a large amount of amino acids, vitamins and the minerals necessary to cultivate microorganisms [2]. Thus substrates of molasses and corn steep liquor may be considered as low-cost alternatives that meet the nutrient and energy (carbon, hydrogen, oxygen and nitrogen) requirements for cultivating any microorganism.

Chitin and chitosan have emerged as among the most promising functional materials to choose from for various modern bio-based industrial applications. For example, they are used by the cosmetics, pharmaceutical and food industries, the agricultural and environmental sectors as well as to treat waste water. Characteristics, such as their being non-toxic, biodegradable, biocompatible, antimicrobially active, environmentally safe and easy to obtain, favor their current and future applicability [12,13].

The antimicrobial activity of chitosan has been pointed out as one of its most promising properties. This activity depends on its molecular weight, degree of deacetylation and the method used to obtain the polymer [14,15]. This is also regarded as one of the most interesting properties of chitosan [16]. Several researchers demonstrated that this polysaccharide has antimicrobial action in a great variety of microorganisms, including gram-positive bacteria and various species of yeast [1,17,18]. Moreover, chitosan has numerous advantages over other chemical disinfectants since it possesses a stronger antimicrobial activity, a broader range of activity, a higher antibacterial activity even at low concentrations, and a lower toxicity towards mammalian cells than other molecules [12].

Therefore, this article puts forward a way to optimize the production of chitin and chitosan by *Cunninghamella elegans* and *Rhizopus arrhizus* using two agroindustrial wastes, namely, corn steep liquor and molasses, as alternative low cost sources of carbon and nitrogen. The physicochemical characteristics and antimicrobial activity of the chitin and chitosan synthesized are also described.

## 2. Results and Discussion

### 2.1. Comparative Influence of Molasses and Corn Steep Liquor on the Production of Biomass, Chitin and Chitosan by C. elegans and R. arrhizus

The influence of different concentrations of molasses and corn steep liquor on the yields of biomass, chitin and chitosan by *C. elegans* and *R. arrhizus* was observed in this study. Table 1 presents the comparative analysis of the results obtained in each assay of the 2^2^ factorial designs. Molasses and corn steep liquor in the highest concentrations presented a directly proportional effect on the increase in biomass production for both fungi. The best yields of biomass by *C. elegans*, 16.00 and 14.47 g·L^−1^, and *R. arrhizus*, 24.60 and 21.00 g·L^−1^, were obtained in Assay 4 (4.00% molasses, 8.00% corn steep liquor) and Assay 6 (2.50% molasses, 5.00% corn steep liquor), respectively, which had the highest concentrations of molasses and corn steep liquor. On the other hand, Assay 2 (4% molasses, 2% corn steep liquor), in which the molasses concentration is twice as high as the corn steep liquor concentration, provided the best yields of chitin by *C. elegans* (72.29 mg·g^−1^) and *R. arrhizus* (83.20 mg·g^−1^). The pure experimental error was calculated from four replicates run corresponding to a central point of the complete factorial (Assays 5–8) as control.

The highest yields of chitosan, 26.29 and 33.13 mg·g^−1^ from *C. elegans*, and 49.31 ^1^ and 40.67 mg·g^−1^ from *R. arrhizus* were presented in culture medium 1, which had the lowest molasses (1%) and corn steep liquor (2%) concentrations, and in the central point with intermediate concentrations (2.5% molasses, 5% corn steep liquor) of these substrates, respectively. Therefore, *R. arrhizus* presented biomass and chitosan yields that were 30% higher compared to those obtained by *C. elegans*. The yield of chitin by *R. arrhizus* was also 13% higher than the yield obtained by *C. elegans*.

There was a decrease in the pH (data not shown) from 6.0 (initial pH) to 5.6 and 4.4 at the end of growth from *R. arrhizus* and *C. elegans* respectively. Similar results were observed by Santos *et al.* [10]. The influence of the culture medium pH in the biomass yield was observed by Nwe *et al.* [19] and Nwe and Stevens [20] who also noted that slightly acidic pH values are more favorable to the fungal growth. The low pH is optimal for the activity of chitin deacetylase and consequently favors the enzymatic deacetylation of chitin into chitosan, thereby increasing the yield of this biopolymer [21].

Bioprocesses are dependent on culture media being under appropriate and favorable conditions for the maintenance of microorganisms if they are to express their biotechnological potential [22]. Therefore, it was the specific concentrations of molasses and corn steep that provided the conditions for obtaining satisfactory yields of biomass, chitin and chitosan by *C. elegans* and *R. arrhizus* when compared to the values presented in the literature (Table 2). The results obtained in this study compared with the yields of biomass, chitin and chitosan by other microorganisms prove that the content of these biopolymers depends on the fungal strains, the age of the mycelia, the culture medium, the growth conditions and the extraction method used for extracting chitin and chitosan [23,24].

The alteration of culture conditions can increase the production of chitin deacetylase, the enzyme responsible for the bio-conversion of chitin to chitosan; and consequently it can influence the synthesis of the cell wall of a fungus and thus improve chitosan productivity [25].

A Pareto chart was used to show the effect of the independent variables of molasses and corn steep liquor on the production of biomass, chitin and chitosan by *C. elegans* and *R. arrhizus*. This statistical analysis has proved effective in assessing the influence of independent variables while seeking to optimize a specific result [5].

**Figure 1.**
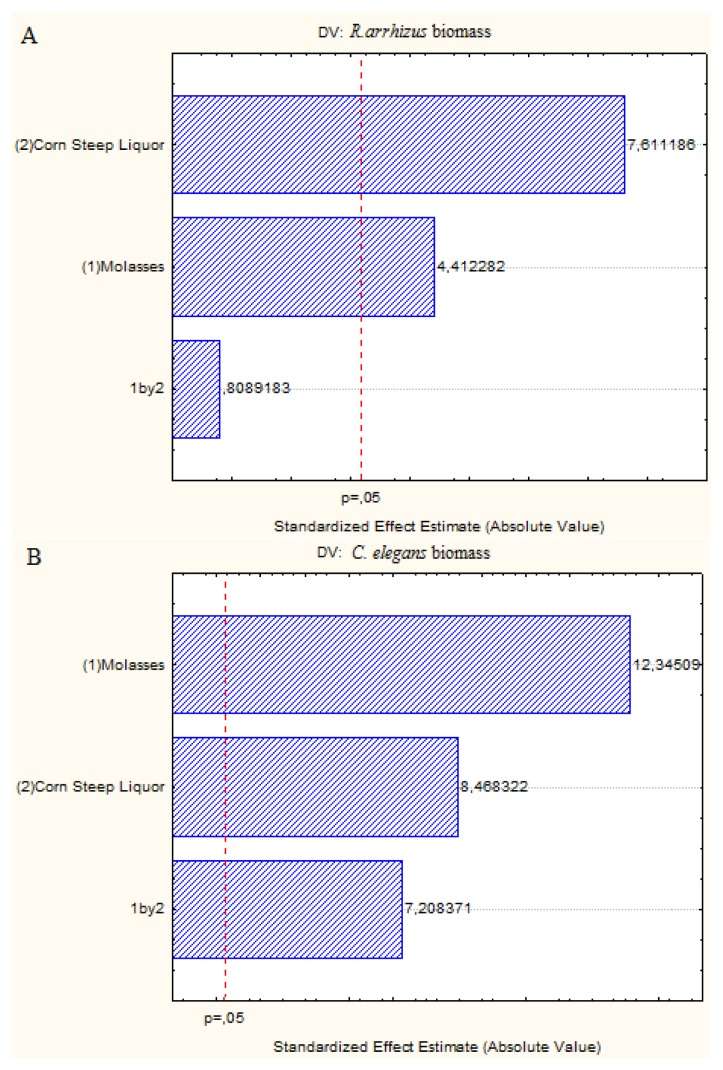
Pareto charts showing the effect of the independent variables, corn steep liquor and molasses, on the biomass production by *R. arrhizus* (**A**) and *C. elegans* (**B**).

The Pareto charts (Figure 1A,B) show the positive effect of the molasses and corn steep liquor, including the interaction of these two independent variables, on the biomass production by both fungi, as also observed in Table 1, Assay 4 (4.00% molasses, 8.00% corn steep liquor) which had the highest concentrations of these variables and biomass production.

**Figure 2.**
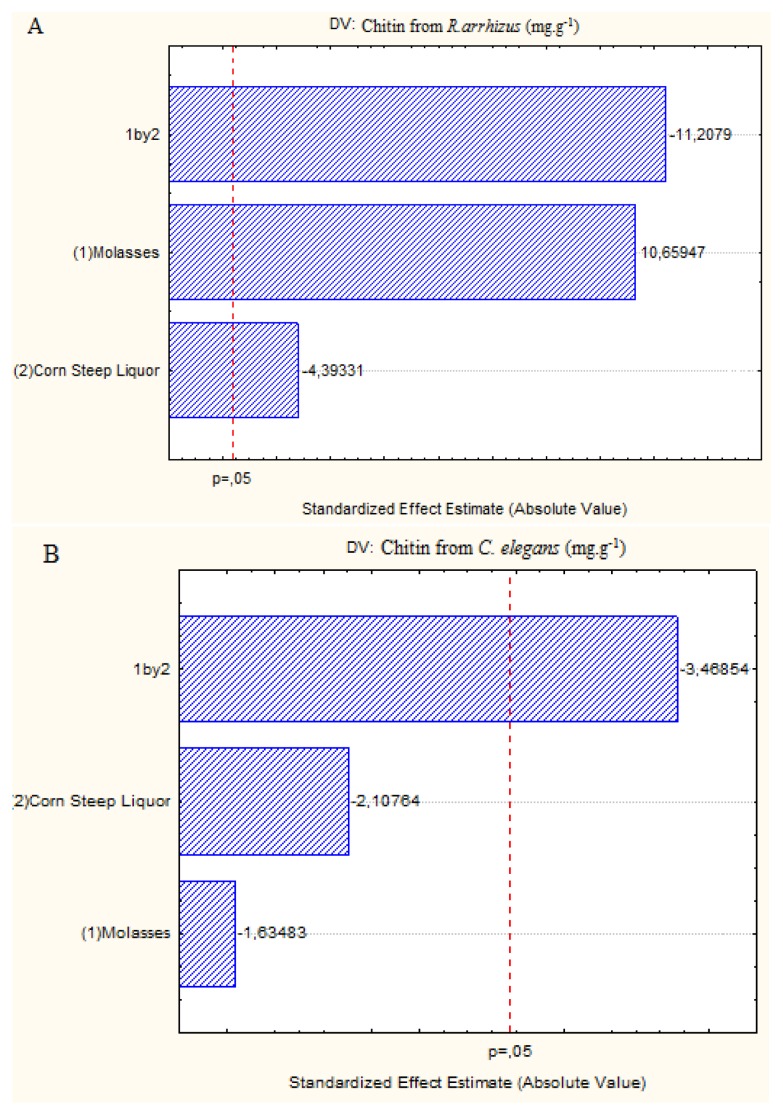
Pareto charts showing the effect of the independent variables, corn steep liquor and molasses, on the chitin yield by *R. arrhizus* (**A**) and *C. elegans* (**B**).

In Figure 2A, the Pareto charts also confirm the positive influence of molasses and the negative influences of corn steep liquor and the interaction between these variables on the production of chitin by *R. arrhizus*. The same result was obtained in Assay 2 (4% molasses, 2% corn steep liquor), which provided the best yields of chitin with only the molasses in the highest concentrations. The Pareto chart, Figure 2B shows that lower concentrations of molasses and corn steep liquor favor the increase of chitin produced by *C. elegans*. The same result occurred in Assays 2 and 3 of the factorial design which presented the highest chitin yields for this fungus grown in low concentrations of corn steep liquor (Assay 2) or molasses (Assay 3).

**Figure 3.**
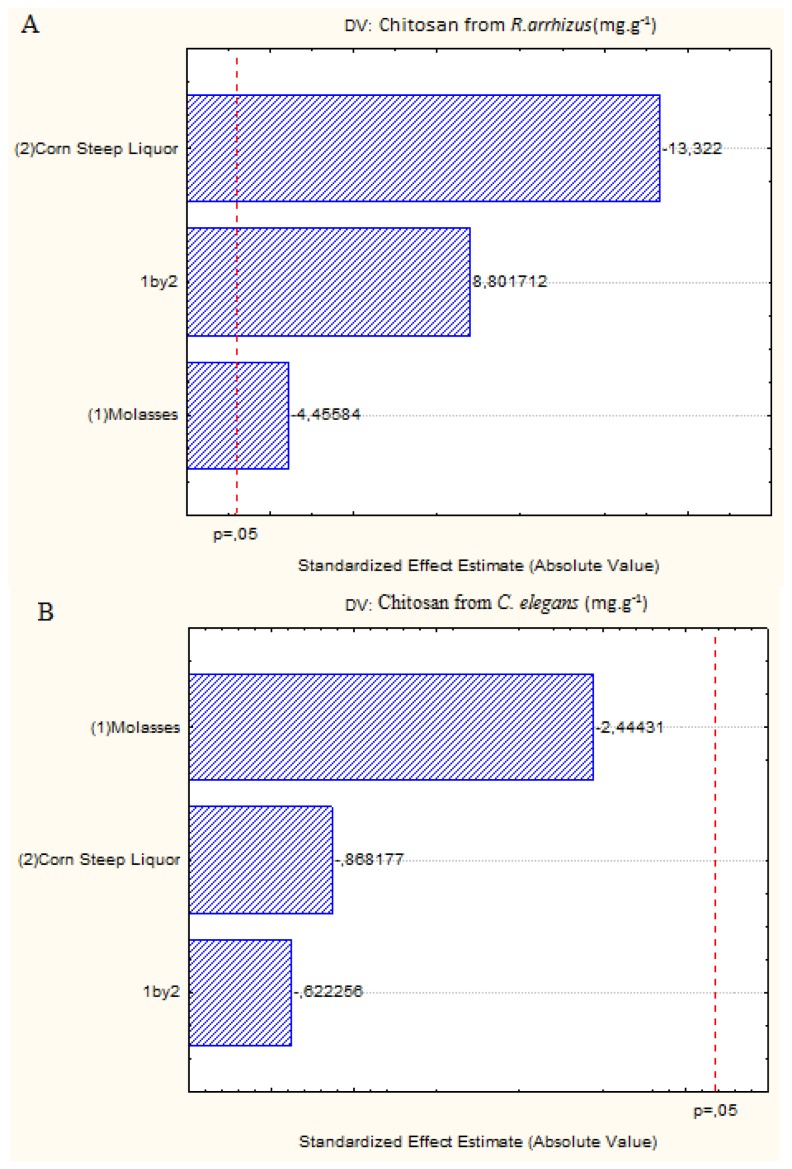
Pareto charts showing the effect of the independent variables, corn steep liquor and molasses, on the chitosan yield by *R. arrhizus* (**A**) and *C. elegans* (**B**).

The results for chitosan shown in Table 1 were also corroborated by Figure 3, which shows the negative effect on chitosan production by *C. elegans* when these substrates are increased. The decrease in the concentrations of molasses or corn steep liquor, or the increase in these two substrates simultaneously in the culture medium promote a higher production of chitosan by *R. arrhizus*. However, higher concentrations of corn steep liquor and molasses than those used at the central point shown do not favor the production of chitosan (Assay 4, Table 1).

**Figure 4.**
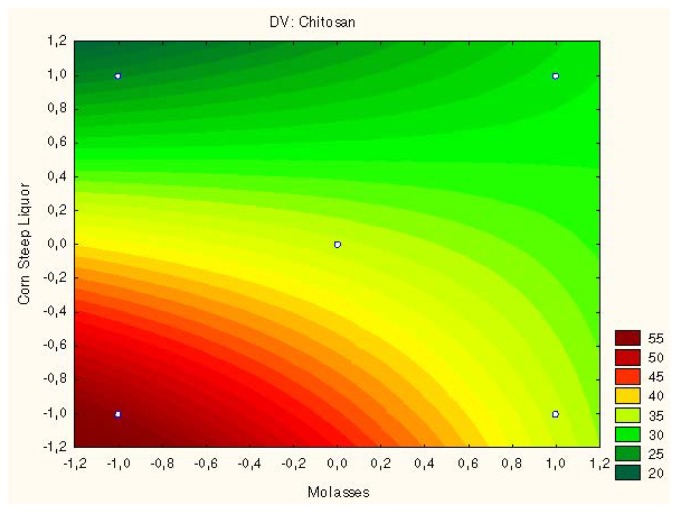
Surface response to chitosan production by *Rhizopus arrhizus* related to interaction of molasses and corn steep liquor.

**Figure 5.**
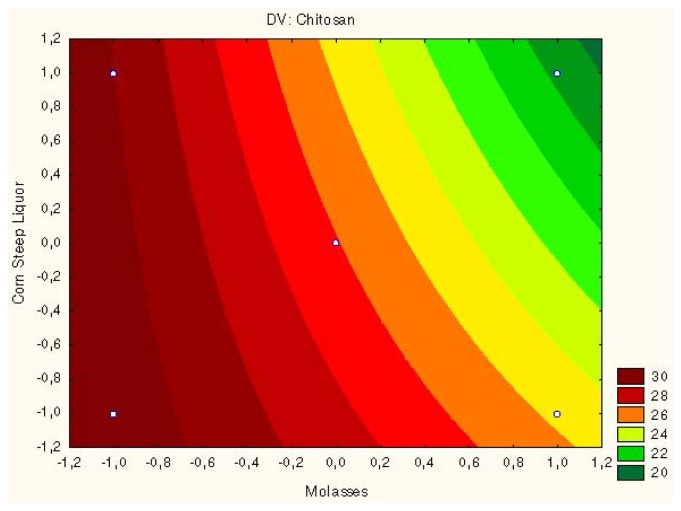
Surface response to chitosan production by *Cunninghamella elegans* related to interaction of molasses and corn steep liquor substrates.

The contour curves (Figures 4 and 5) showed the optimize chitosan production by *Rhizopus arrhizus* and *Cunninghamella elegans* using corn steep liquor of (2%) and molasses (1%) medium.

The analysis of these results suggests that there is an economic culture medium, with specific concentrations of molasses and corn steep liquor as sources of carbon and nitrogen, for obtaining better yields of biomass, chitin and chitosan by *C. elegans* and *R. arrhizus*. Thus, probably to obtain better biomass production by these fungi it would be necessary to increase the concentration of molasses and corn steep liquor. From these fungi, greater chitin yields could be obtained using a culture medium with higher concentrations of molasses and lower concentrations of corn steep liquor. Furthermore, an increase in chitosan production could be achieved using values of molasses or corn steep liquor below the lowest levels of these substrates tested or intermediate concentrations. These large influences of culture conditions and nutritional sources for obtaining satisfactory yields of microbial chitosan were also observed by Pochanavanich and Suntornsuk [24] and Nadarajah *et al.* [27].

Other studies also show increased production of biomass, chitin and chitosan by Mucoralean fungi such as *Absidia corymbifera* [25], *R. arrhizus* [2,25,28], *C. elegans* [10] and *Syncephalastrum racemosum* [9] in culture medium with specific concentrations of corn steep liquor. Santos *et al.* [10] reported that the increase in biomass production by *C. elegans* is due to the presence of considerable amounts of amino acids (alanine, arginine, histidine, leucine, lysine, tyrosine, phenylalanine) and vitamins (biotin, choline, inositol, niacin, pyridoxine, thiamine) that are essential for the growth of the microorganism. Edwinoliver *et al.* [29] also suggest that the use of corn steep liquor for lipase production from *Aspergillus niger* makes the process green, because this substrate is renewable and economically viable on an industrial scale.

Amorim *et al.* [11] also showed that molasses is an inexpensive carbon source for the growth and production of chitosan by *Cunninghamella bertholletiae* and they suggest the cultivation of this fungus by using only these carbon sources without nitrogen supplements will result in satisfactory growth and chitosan production. However they have also shown that high concentrations of molasses inhibit the production of chitosan, as observed in this study. Amorim *et al.* [11] suggested that the concentration of sugar was not the only factor responsible for the decrease of chitosan in a medium of molasses. Probably this could be explained by the presence of other substances generated during different production processes which inhibited the enzyme that produces chitosan, as shown by the highest quantities of inorganic impurities detected by thermogravimetric analyses in chitosan preparations obtained from the growth of *C. bertholletiae* cells in molasses.

The controlled use of low cost substrates for replacing or supplementing culture media, generally used with commercial nutrients, can decrease the final value of byproducts, mainly in large scale production, and in addition this alternative promotes the recovery of environments contaminated by agribusiness. This inexpensive source also offers promising advantages for industrial scale production over the chitosan obtained from crustacean shells, because of less use of solvents, heat treatments and simultaneous extraction of chitin and chitosan during the extraction process. The adequate control of the fermentative process can offer a polymer production from fungal biomass continuously, in a short time, with minimum substrate consumption.

### 2.2. Characterization of Chitin and Chitosan Extracted from C. elegans and R. arrhizus

#### 2.2.1. Infrared Spectroscopy (Deacetylation Degree—DD%)

**Figure 6.**
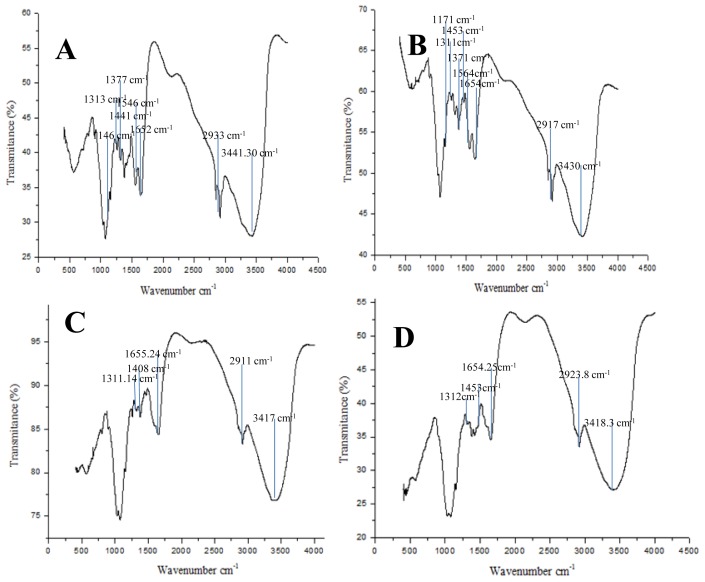
Infrared absorption spectra: (**A**) Chitosan produced by microbiological *R. arrhizus* Assay 7; (**B**) Commercial chitosan (Sigma Aldrich Corp., St. Louis, MO, USA); (**C**) Chitin produced by microbiological *R. arrhizus* Assay 2; (**D**) Commercial chitin (Sigma).

The infrared spectrum of chitin and chitosan from *R. arrhizus* (Figure 6) grown in the culture medium of Assays 1 and 2 were similar to those reported in the literature [5,8,30]. The chitin from *R. arrhizus* showed the presence of two types of amide group (amide I and II). The most significant parts of both chitins were the characteristic bands related to the CN bond stretching plus CH_3_ wagging (1311 and 1313 cm^−1^); the N–H deformation in the CONH plane, including amide II to (1546 and 1564 cm^−1^); and the carbonyl group stretching, C=O (amide I) (1652 and 1654 cm^−1^). In a similar way, chitin shows the specific bands in the amide II region: 1146 and 1171 cm^−1^; 1371 and 1377 cm^−1^ (C–O stretching of the –CH_2_–OH group); 1441 and 1453 cm^−1^, the axial deformation of the amide C–N; 2933 and 2917 cm^−1^, assigned to the C–H stretching; and 3441 and 3430 cm^−1^, corresponding to the axial deformation of OH, which appears overlapping the band of the axial NH deformation.

The infrared spectrum of chitosan from *R. arrhizus* also presented the most significant amide bands namely 1312–1311, 1453–1408, and 1654–1655 cm^−1^, but these peaks are less intense than in chitin, mainly the peak of 1654–1655 cm^−1^. When compared with the infrared spectrum of chitin, the chitosan spectrum showed the band disappearing at 1550 cm^−1^ (amide II vibrational mode) and the progressive weakening of the band 1655 cm^−1^ (amide I vibrational mode) as a consequence of the *N*-deacetylation process. Chitosan also presented the bands 2920.97 and 3420.99 cm^−1^, as observed in the spectrum of chitin obtained in this study. Ebrahimzadeh *et al.* [30] related that during chitosan production, acetyl is eliminated after hydrolysis and, consequently, the carbonyl band is eliminated in chitosan. However, the infrared spectrum of chitosan from fungal with these amide bands, *i.e.*, the acetyl in the amino group (stretching, C=O, amide I) shows that the chitosan is not completely deacetylated.

The Degree of Deacetylation (DD) is an important parameter associated with the physico-chemical properties of chitosan, because it is linked to the cationic properties of chitosan [4,8,24]. In this article, chitin (Assay 2) presented a DD of 25% and 40% and chitosan a DD of 80% (Assay 7) and of 82% (Assay 1) from *C. elegans* and *R. arrhizus*, respectively which are similar to the DDs reported in the literature [11,23,31].

#### 2.2.2. Viscosity and Molecular Weight

The viscosity and molecular weight of fungal chitosan were 3.40 centipoises and 4.96 × 10^3^ g·mol^−1^ from *R. arrhizus* and 3.79 (cP) and 5.08 × 10^3^ g·mol^−1^ from *C. elegans* respectively. The result is in agreement with the literature, which reports molar weights ranging from 1.0 × 10^3^ to 9.0 × 10^5^ g·mol^−1^ [2,11]. The method for chitin and chitosan extraction used in this study with high temperatures and NaOH solution may have influenced the breaking of the polymer resulting in chitosan of a lower molecular weight. These results are considerably lower than the viscosity of crab chitosan and similar to other fungal chitosans [24]. Some authors report the viscosity of fungal chitosan from 2.7 to 11.3 cP [30,32] and that of crab shell chitosan between 316.2 and 372.7 cP [24].

The viscosity of chitosan is directly proportional to its molar mass and the results presented in this paper corroborate this statement. The chitosan isolate showed low viscosity and also exhibited lower molar mass, suggesting the molecular weight of fungal chitosan may be lower than that of crab chitosan. These characteristics provide improved solubility in water at physiologically acceptable pH values which facilitates some applications in the food, medical, agricultural industries as an antimicrobial and preservative agent according to the literature [24,32,33]. Tayela *et al.* [15] showed that the most bioactive chitosan type for inhibiting the growth of *Candida albicans* showed the lowest molecular weight (32 kDa) and the highest degree of deacetylation (94%).

#### 2.2.3. X-ray Diffraction

X-ray diffraction is commonly used to determine the polymorphic forms of a compound which has different crystalline structures for which distinct powered X-ray diffraction patterns are obtained [4,31]. Based on these patterns it is possible to observe differences in the spacing of crystal planes and the polymorphic structure, and to provide accurate measurements of the crystalline content, which influences the physical and biological properties of the polymer [4,13,31].

**Figure 7.**
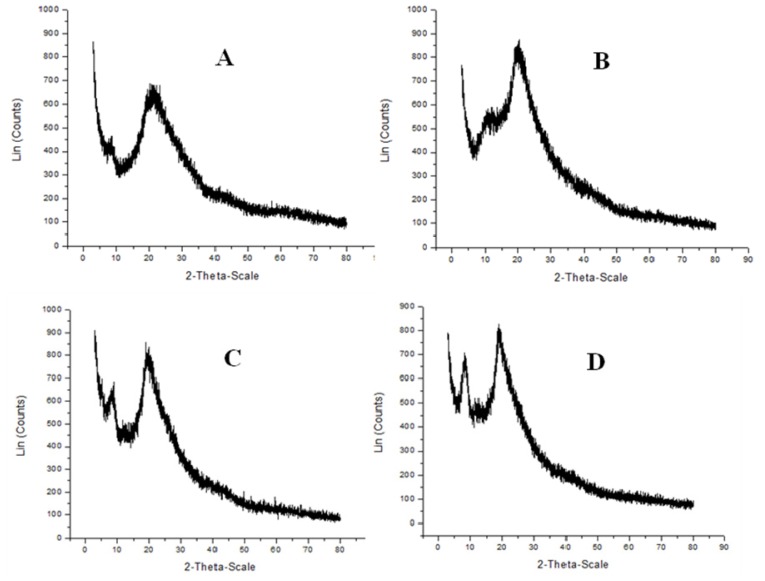
X-ray diffractograms of chitin (**A**,**B**) and chitosan (**C**,**D**) obtained from *C. elegans* and *R. arrhizus* biomass, respectively.

Figure 7 shows the X-ray diffractograms of chitin and chitosan by *C. elegans* and *R. arrhizus* in Assays 1 and 2 of the factorial design. The crystallinity indices of these biopolymers were determined from the scattering intensity at two angles, one at 2θ *=* 9°–10°, representing the diffraction intensity of amorphous regions and another at 2θ *=* 19°–20°, the diffraction intensity of the crystalline regions. This peak at about 9° disappeared in both fungal chitins. Probably this suggests a biopolymer with a more crystalline structure. This could also indicate the need of a purification process to obtain a satisfactory biopolymer [34]. The diffraction pattern of chitosan (Figure 5C,D) by *C. elegans* in Assay 2 (Table 3) showed strong Bragg refractions at angles of 20.0° and 9.0° which are two characteristic peaks of chitosan, and are similar to those given in the literature [4,7].

The results from the crystallinity indices were 33.80% and 32.8% for chitin, and 20.30% and 17.8% for the chitosan obtained by *C. elegans* and *R. arrhizus* in Assay 2. Overall, the crystallinity index of chitin was higher than that of chitosan, in which a lower crystallinity of polysaccharides indicates disruption of intra- and inter-molecular hydrogen bonds. The higher crystalline index of chitin reflected: its higher degree of crystallinity and its more ordered structure and the lower crystallinity of chitosan indicated disruption of intra- and inter-molecular hydrogen bonds [35]. These results were also lower than those obtained for chitin (45.60%) and chitosan (23.82%) from the crustacean standard (data not shown). The results are also supported in the literature [4,31]. Tolaimate *et al.* [36] suggested that these lower crystallinity indices in chitin and chitosan produced by microbial fermentation might indicate their improved water solubility in comparison with chitin and chitosan prepared from the chemical extraction method, probably due to the more severe extraction conditions during chemical extraction. Some authors also related that the crystallinity index of chitosan is related to its DD function [4,31].

#### 2.2.4. Scanning Electron Microscopy

**Figure 8.**
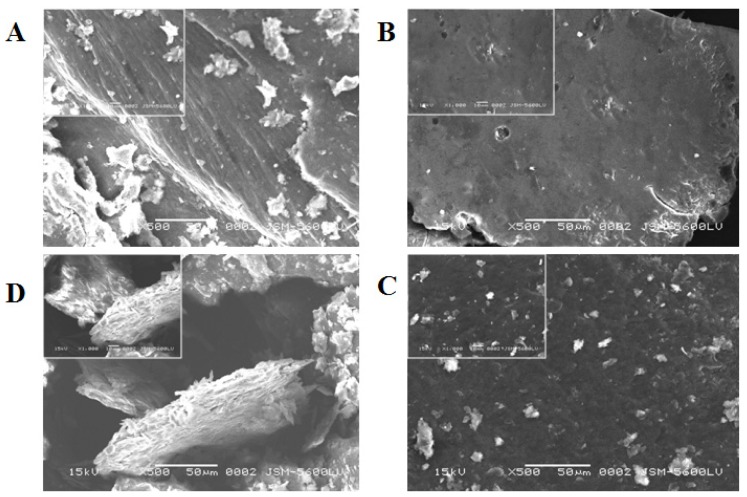
Scanning electron microscopy (SEM) photographs of chitin (**A**) and chitosan (**B**) produced by *R. arrhizus* and chitosan (**C**) and chitin (**D**) produced by *C. elegans*, in Assays 1 and 2, at 500× and 1000× magnification. The measurement bar = 50 μM.

The chitin and chitosan produced by *C. elegans* and *R. arrhizus* in Assays 1 and 2 were selected for examination by scanning electron microscopy (Figure 8).

The chitin showed a prominent arranged microfibrillar crystalline structure in SEM (Figure 6A,D) which was absent in the chitosan (Figure 6B,C), as observed by Yen *et al.* [37], Arbia *et al.* [5]; and Chan, Chen, and Yuan [38], Yen and Mau [39]. The crystallinity of fungal chitin observed is reinforced with the prominent arranged microfibrillar crystalline structure of this biopolymer in SEM (Figure 5A,D).

Yen *et al.* [37] related that the crystallinity structure observed between fungal and crab chitins might also be attributed to their different intersheet or intrasheet hydrogen-bonding systems. However, the preview of this microfibrilar structure in the chitin may have arisen from the extraction process of this biopolymer, *i.e.*, the deproteinization step. The fungal chitosan exhibited a more compact, denser structure, with layers of crumbling flake without porosity as observed in crustacean chitosan by Yen *et al.* [37] and Yen and Mau [39] for fungal chitosan.

### 2.3. Antimicrobial Activity

The effectiveness of chitosan from *C. elegans* and *R. arrhizus* in inhibiting the growth of *Listeria monocytogenes*, *Staphylococcus aureus*, *Pseudomonas aeruginosa*, *Salmonella enterica*, *Escherichia coli*, and *Yersinia enterocolítica* by determining the MIC and MBC is shown in Table 3. Both chitosans showed identical MIC and MBC for all bacteria assayed. All bacteria grown in presence of acetic acid 1%. These results are in agreement with those reported in the literature [40,41]. The chitosan samples tested were more effective at inhibiting Gram negative bacteria compared to gram positive ones, except for *L. monocytogenes*, which showed a higher MIC and MBC.

The antimicrobial activity of chitosan is well documented against a number of pathogenic microorganisms, with MIC varying from 0.01% to 1% [41,42]. The antibacterial activity of chitosan *in vitro* depends on the physicochemical characteristics of chitosan and the species, or even the strain of the bacteria tested [14,43].

Chung *et al.* [14] investigated the relation between antimicrobial activity of chitosan and the characteristics of the cellular wall of bacteria. The authors verified that chitosan is more efficient as an antibacterial agent against gram-negative bacteria due to the composition of phospholipids and carboxylic acids of the bacterial cellular wall. These results suggest that the effects of chitosan are distinct in the two types of bacteria: in the case of gram-positive ones, the hypothesis is that chitosan of low molecular mass penetrates into bacteria, causing ruptures in the metabolism of these microorganisms. In addition these authors demonstrated that although the hydrophilicity of the cell wall is similar among gram-negative bacteria, the distribution of negative charges on their cell surfaces can be quite different. Most negatively charged cell surfaces have a greater interaction with chitosan. This could explain why the susceptibility of *L. monocytogenes*, *S. enterica* and *Y. enterocolitica* to both microbial chitosans in this study were different from those of the other gram-negative bacteria tested (Table 3).

Chitosan exhibited antibacterial activity only in an acidic medium, which was usually attributed to the poor solubility of chitosan above pH 6.5 and its more positively charged polycationic molecules with stronger affinity for cells [44,45]. Therefore, for the antimicrobial activity, chitosan was dispersed in a solution of 1% acetic acid. As acetic acid itself has antibacterial activity, a positive control was used, whereby chitosan was replaced with sterile distilled water and 1% acetic acid, for each microorganism to assure that the antimicrobial activity evidenced is attributed to chitosan. Microbial growth was observed in all positive controls. In addition, the viability of the bacterial strains was confirmed by verifying their growth in Brain Heart Infusion (BHI) agar without adding chitosan.

Studies with electronic micrographs demonstrate that in gram-positive bacteria the chitosan weakens or even breaks up the bacterial cellular wall, while in gram-negative bacteria, the cytoplasm is concentrated and the cell interstice is extended. The external membrane of the cellular wall of the gram-negative bacteria consists of lipopolysaccharides (LPS) that provide a hydrophilic surface for the bacterium. The LPS also have anionic groups (phosphate, carboxyl), which contribute to the stability of the LPS through electrostatic interactions with divalent cations. The removal of these cations by chelant agents results in the run down and molecule release of the LPS. On the other hand, the cellular wall of the Gram-positive bacteria consists mainly of peptidoglican (PG) and teichoic acid—TA (polymer polyanion), which are linked covalently to the acid Nacetylmuramic of the PG or anchored in the direction of the cytoplasmic membrane, via glycolipid (lipoteichoic acid—LTA), which provides a binding site with the chitosan, causing functional ruptures in the membrane [1,18].

## 3. Experimental Section

### 3.1. Materials

All reagents used were of analytical grade. The acetic acid and sodium hydroxide were obtained from Vetec (São Paulo, Brazil). The molasses from sugar cane was procured from a local market and corn steep liquor, a byproduct of corn manufacturing industry, was kindly donated by Corn Products do Brasil, Cabo de Santo Agostinho, PE, Brazil. These agroindustrial wastes were used as soluble substrates and the carbon and nitrogen sources in the 2^2^ factorial design.

### 3.2. Microorganisms and Biomass Production to Obtain Chitin and Chitosan

*C. elegans* strain UCP/WFCC 0542 and *R. arrhizus* UCP/WFCC 0402 isolated from mangrove sediments situated in Rio Formoso, PE, Brazil were used. These strains were deposited in the Culture Collection of the Catholic University of Pernambuco, Brazil, registered in the World Federation for Culture Collection (WFCC). The fungi were maintained on Potato Dextrose Agar (PDA) medium at 5 °C and transferred to a new medium every four months.

For biomass production, both fungi were grown in Petri dishes containing PDA medium at 28 °C for 8 days until sporulation. Petri dishes containing PDA were inoculated with 1 mL of the sporangiole suspension (10^7^ spores·mL^−1^) of *C. elegans* and *R. arrhizus* and maintained for 18 h at 28 °C. After the incubation time, 20 discs (1 cm diameter) with mycelium of both fungi were cut from these Petri dishes and inoculated in an Erlenmeyer flask containing 200 mL of the alternative medium with molasses and corn steep liquor, pH 5.6. The flasks were incubated at 28 °C in an orbital shaker at 150 rpm, for 96 h. The mycelia were harvested, washed twice in distilled water by filtration, using a nylon membrane silkscreen (120 F). After lyophilization, the biomass was maintained in a vacuum dessicator until constant weight.

### 3.3. Factorial Design

A 2^2^ full factorial design was carried out to analyze the main effects and interactions of molasses (1%–4%) and corn steep liquor (2%–8%) on the response variable of biomass, chitin and chitosan yield by *C. elegans* and *R. arrhizus* to select the best condition for producing mycelia and biopolymers in accordance with the variables established (Table 4). Pareto charts were compiled to validate the influence between these agroindustrial wastewaters (independent variables) and the response variables. An estimate of pure experimental error was calculated from four replicates run corresponding to a central point of the complete factorial. The data obtained from the experiments were subjected to statistical analysis by STATISTICA software version 7.0 (StatSoft Inc., Tulsa, OK, USA) and the significance of the results was tested at the *p* < 0.05 level.

### 3.4. Chitin and Chitosan Extraction

Chitin and chitosan were extracted using dry biomass of *C. elegans* and *R. arrhizus* as described by Hu *et al.* [46]. The lyophilized biomass was deproteinized with 1 M NaOH solution (1:30 *w*/*v*, 121 °C, 15 min). The alkali-insoluble fraction was separated by centrifugation (4000× *g*, 20 °C, 10 min), and treated using 2% of acetic acid (1:30 *w*/*v*, 100 °C, 15 min) followed by centrifugation at 4000× *g*, 20 °C, 15 min. Chitin was considered an acid insoluble material, and the supernatant was alkalized to pH 10, maintained overnight at 5 °C and centrifuged (4000× *g*, 20 °C, 10 min) so as to precipitate the chitosan. The chitin and chitosan were washed with distilled water four times, freeze-dried, and kept in a dessicator until constant weight.

### 3.5. Characterization of Chitin and Chitosan from C. elegans and R. arrhizus

#### 3.5.1. Infrared Spectroscopy (Degree of Deacetylation—DD%)

The degree of deacetylation (DD%) for microbial chitin and chitosan was determined using infrared spectroscopy as per Baxter *et al.* [47], using the absorbance ratio A1655/A3450 and calculated as per Equation (1):

(1) DD (%)=100-[(A1655/A3450)×115]

A two-milligram sample of fungal chitin and chitosan, which had been dried overnight at 60 °C under reduced pressure was thoroughly blended with 100 mg of KBr to produce 0.5 mm thick disks. The disks were dried for 24 h at 110 °C under reduced pressure. An infrared spectrometry reading was taken with a Bruker 66 Spectrometer (Bruker Corporation Inc., Billerica, MA, USA), using 100-mg KBr disks for reference.

#### 3.5.2. Viscosity and Molecular Weights of Chitosan

The viscosity of 1% chitosan in buffer solution (acetic acid/sodium acetate, pH ~4.5), was determined using a Brookfield digital Rheometer (Model DV-II, Brook Engineering laboratories, Inc., Stoughton, MA, USA) at 25 °C, Spindle CPE-40, 0.5 mL sample volume [8].

The molecular weight of chitosan was determined using an AVS-350 viscometer (Schott-Geräte, Quebec City, QC, Canada), type/capillary: Cannon-Fenske *d*_inside_ = 1.01 mm, at 25 °C. After obtaining the intrinsic viscosity from tables, *K* and *a*, were obtained for HAc/NaAc. *K* = 0.076, *a* = 0.76. The flow time was determined in seconds. Using the Mark-Houwink equation, the average viscosimetric molecular weight was expressed in g·mol^−1^ [48]. See Equation (2):

(2) $$[\eta ]=K{\left({\bar{M}}_{v}\right)}^{a}$$

#### 3.5.3. Crystallinity Index

The X-ray diffractograms of chitin and chitosan were obtained in the X-ray Laboratory of the Physics Department Federal University of Pernambuco—UFPE. The measurement was taken using SIEMENS Model 5000 D X-ray equipment (Siemens Corporation, Aubrey, TX, USA), Cu Kα radiation with λ = 1.542 Å, in a scanning range between 4° and 50° with a rate of 0.02 min^−1^. The interplanar distance was determined by the half height width of the peak of greatest intensity (IC). The crystallinity index (ICR) was determined using the following equation:

(3) $$\text{Crystallinity index }(\%)=100\{[I(\theta_{\text{c}})-I(\theta_{\text{a}})]/I(\theta_{\text{c}})\}$$

where *I*(θ_c_) is the relative intensity of the crystalline regions (2θ = 20°) and *I*(θ_a_) corresponds to amorphous regions (2θ = 9°) for chitosan.

#### 3.5.4. Scanning Electron Microscopy

The dried sample was ground under vacuum using a sputter coater and its surface was observed using a scanning electron microscope Series XL 30 (Umax) ESEM (Env. Scan. Electron Micros, Jeol, Tokyo, Japan) with tungsten filament, at 20 kV accelerating voltage.

### 3.6. Bacterial Strains and Culture Conditions for the Antimicrobial Assay

*L. monocytogenes* ATCC 7664, *P. aeruginosa* ATCC 9027, *S. enterica* ATCC 6017, and *Y. enterocolítica* ATCC 9610 for antimicrobial assay were provided by FIOCRUZ, Rio de Janeiro, Brazil. *S. aureus* ATCC 6538, *E. faecalis* ATCC 29212 and *E. coli* ATCC 8739 were donated by the Antibiotics Institute, UFPE (Recife, Brazil). Stock cultures were kept on Muller Hinton agar slants with blood added (5% *v*/*v*) at 4 °C. Inoculums used in the experimental assays were obtained from cultures grown overnight (18 h) in Brain Heart Infusion broth (DIFCO Laboratories, Detroit, MI, USA) at 37 °C. After incubation, the bacterial cells were separated from the growth medium by centrifugation at 10,000× *g* for 15 min at 4 °C, washed thrice in buffer KCl (0.05 M KCl, 1.0 mM KH_2_PO_4_, 1.0 mM CaCl_2_, 0.1 mM MgCl_2_, pH 6.0), and resuspended in buffer KCl. Suspensions were adjusted so that the optical density (OD) at 660 nm was 1.5, which provided bacterial inoculum of approximately 5 × 10^8^ Colony Forming Unity per mL (CFU·mL^−1^).

### 3.7. Chitosan Solution Preparation

Chitosan from *C. elegans* and *R. arrhizus* were solubilized in a solution of 1% acetic acid at concentrations to 20 mg/mL (*w*/*v*). The pH of the solutions were adjusted to pH 5.8 using HCl and NaOH.

### 3.8. Antimicrobial Activity

Minimum inhibitory concentration (MIC) and minimum bactericidal concentration (MBC) of chitosan on the assayed bacteria were carried out using the broth dilution method (Heilman test) as described by Chambrevil; Marmonier [49]. For this, a 0.1 mL aliquot of bacterial inoculum was inoculated into screw-capped 13 × 130 mm sterile tubes containing 0.9 mL of BHI broth containing the desired chitosan concentration (5000–50 μg·mL^−1^) followed by shaking using Vortex for 30 s. The system was incubated at 37 °C for 24 h and the MIC was defined as the lowest chitosan concentration providing no visible growth (turbidity) and the MBC was the lowest chitosan concentration able to cause a 99.9% kill rate of the initial inoculum. MBC was found by inoculating a 25-μL aliquot of the chitosan-treated Assay into sterile Muller Hinton agar Petri dishes and was followed by incubation at 37 °C for 48 h. For positive control, chitosan was replaced with sterile distilled water and 1% acetic acid. The assays were conducted in triplicate and the results expressed as average values. Also, the viability of the bacterial strains was assessed by verifying their capacity to grow in a Muller-Hinton agar without adding chitosan.

## 4. Conclusions

These results suggest a new economic culture medium to improve chitin and chitosan production from mycelial biomass by *C. elegans* and *R. arrhizus* with a low molecular weight and degree of deacetylation of approximately 80%. The data obtained from this research point out that the selection of chitosan is one of the important factors to consider before applying chitosan in medical sectors, considering the antimicrobial potential of chitosan against pathogenic bacteria. Therefore chitosan shows great promise as an alternative natural antimicrobial agent against gram-positive and gram-negative bacteria. All in all, it is expected that much progress will be made in reaching the final goal of developing the mechanism of chitosan against bacterial pathogens at the molecular level.

## Figures and Tables

**Table 1.** Biomass, chitin and chitosan produced by *C. elegans* and *R. arrhizus* in each assay with different concentrations of molasses and corn steep liquor varied symmetrically around the central point according to the 2^2^ factorial designs.

Assays	Biomass (g·L^−1^)		Chitin (mg·g^−1^)		Chitosan (mg·g^−1^)	

	*C. elegans*	*R. arrhizus*	*C. elegans*	*R. arrhizus*	*C. elegans*	*R. arrhizus*
1	7.41	8.25	64.96	45.72	26.29	49.31
2	9.53	13.15	72.29	83.20	21.40	31.83
3	7.93	17.50	70.40	57.40	25.63	20.14
4	16.00	24.60	50.00	56.46	17.40	25.87
5	14.47	18.00	59.09	73.29	28.33	37.67
6	13.61	21.00	63.00	70.48	26.84	40.67
7	13.58	20.60	69.11	70.53	33.13	39.00
8	13.90	19.30	60.40	69.23	29.43	38.15

**Table 2.** Biomass, chitin and chitosan production by *C. elegans* and *R. arrhizus* grown on agroindustrial waste compared with results obtained by other studies in the literature.

Microorganism	Substrate	Biomass (g·L^−1^)	Chitin (mg·g^−1^)	Chitosan (mg·g^−1^)	Reference
*C. elegans*	Corn steep liquor and molasses	16.00	72.29	33.13	This study
*R. arrhizus*	Corn steep liquor and molasses	24.60	83.20	49.31	This study
*R. arrhizus*	Corn steep liquor and cassava wastewater	8.80	54.38	20.51	[2]
*C. elegans*	Coconut water	2.19	389	129	[2]
*R. arrhizus*	Corn steep liquor 4%	13.00	30.40	12.85	[26]
*Mucor circinelloides*	Yam bean	20.70	500	64	[4]
*C. elegans*	Yam bean	24.30	440	66	[8]
*Absidia corymbifera*	Candy effluent, corn steep liquor	12.68	12.89 (%) 128.9 mg/g	52.71 (%) 520 mg/g	[22]

**Table 3.** Minimum inhibitory concentration (MIC) and minimum bactericidal concentration (MBC) of chitosan from *C. elegans* and *R. arrhizus* against food pathogenic and spoilage bacteria.

Microorganism	*C. elegans*		*R. arrhizus*	

	MIC	MBC	MIC	MBC
*S. aureus*	300 μg·mL^−^	500 μg·mL^−^	300 μg·mL^−^	500 μg·mL^−^
*E. faecalis*	400 μg·mL^−^	600 μg·mL^−^	400 μg·mL^−^	600 μg·mL^−^
*E. coli*	200 μg·mL^−^	400 μg·mL^−^	200 μg·mL^−^	400 μg·mL^−^
*P. aeruginosa*	200 μg·mL^−^	400 μg·mL^−^	200 μg·mL^−^	400 μg·mL^−^
*L. monocytogenes*	500 μg·mL^−^	1000 μg·mL^−^	500 μg·mL^−^	1000 μg·mL^−^
*Y. enterocolítica*	300 μg·mL^−^	600 μg·mL^−^	300 μg·mL^−^	600 μg·mL^−^
*S. enterica*	300 μg·mL^−^	500 μg·mL^−^	300 μg·mL^−^	500 μg·mL^−^

**Table 4.** Design matrix for the factorial experiments used to evaluate the influence of two factors (molasses and corn steep liquor) on biomass, chitin and chitosan production by *C. elegans* UCP/WFCC 0542 and *R. arrhizus* UCP 402/WFCC, with experimental conditions set at the average of two extreme levels.

Assays	Factor Levels	

	Molasses 1	Corn Steep Liquor 2
1	−1	−1
2	+1	−1
3	−1	+1
4	+1	+1
5	0	0

1 Concentration of Molasses (%, *v*/*v*): 1.00 at level −1; 2.50 at level 0; 4.00 at level +1;

2 Concentration of corn steep liquor (%, *v*/*v*): 2.00 at level −1; 5.00 at level 0; 8.00 at level +1.
